# Chp8, a Diguanylate Cyclase from *Pseudomonas syringae* pv. Tomato DC3000, Suppresses the Pathogen-Associated Molecular Pattern Flagellin, Increases Extracellular Polysaccharides, and Promotes Plant Immune Evasion

**DOI:** 10.1128/mBio.01168-14

**Published:** 2014-05-20

**Authors:** Christoph Engl, Christopher J. Waite, Joseph F. McKenna, Mark H. Bennett, Thorsten Hamann, Martin Buck

**Affiliations:** ^a^ Department of Life Sciences, Imperial College London, London, United Kingdom;; ^b^ Department of Biological and Medical Sciences, Oxford Brookes University, Oxford, United Kingdom;; ^c^ Department of Biology, Norwegian University of Science and Technology, Trondheim, Norway

## Abstract

The bacterial plant pathogen *Pseudomonas syringae* causes disease in a wide range of plants. The associated decrease in crop yields results in economic losses and threatens global food security. Competition exists between the plant immune system and the pathogen, the basic principles of which can be applied to animal infection pathways. *P. syringae* uses a type III secretion system (T3SS) to deliver virulence factors into the plant that promote survival of the bacterium. The *P. syringae* T3SS is a product of the hypersensitive response and pathogenicity (*hrp*) and hypersensitive response and conserved (*hrc*) gene cluster, which is strictly controlled by the codependent enhancer-binding proteins HrpR and HrpS. Through a combination of bacterial gene regulation and phenotypic studies, plant infection assays, and plant hormone quantifications, we now report that Chp8 (i) is embedded in the Hrp regulon and expressed in response to plant signals and HrpRS, (ii) is a functional diguanylate cyclase, (iii) decreases the expression of the major pathogen-associated molecular pattern (PAMP) flagellin and increases extracellular polysaccharides (EPS), and (iv) impacts the salicylic acid/jasmonic acid hormonal immune response and disease progression. We propose that Chp8 expression dampens PAMP-triggered immunity during early plant infection.

## IMPORTANCE

The global demand for food is projected to rise by 50% by 2030 and, as such, represents one of the major challenges of the 21st century, requiring improved crop management. Diseases caused by plant pathogens decrease crop yields, result in significant economic losses, and threaten global food security. Gaining mechanistic insights into the events at the plant-pathogen interface and employing this knowledge to make crops more resilient is one important strategy for improving crop management. Plant-pathogen interactions are characterized by the sophisticated interplay between plant immunity elicited upon pathogen recognition and immune evasion by the pathogen. Here, we identify Chp8 as a contributor to the major effort of the plant pathogen *Pseudomonas syringae* pv. tomato DC3000 to evade immune responses of the plant.

## INTRODUCTION

According to recent estimates by the Food And Agriculture Organization of the United Nations, the global demand for food is projected to rise by 50% by 2030 (1). Meeting this increasing need will be one of the major challenges of the 21st century. Diseases caused by plant pathogens represent a large agricultural burden. They decrease crop yields, resulting in significant economic losses, and threaten global food security (2, 3). Thus, by gaining mechanistic insights into the events at the plant-pathogen interface and employing this knowledge to make crops more pathogen resilient, strategies for improving crop management can be developed.

The bacterial plant pathogen *Pseudomonas syringae* infects more than 50 different cultivars, resulting in diseases such as bacterial speck, brown spot, halo blight, olive knot, wildfire, or bleeding canker in economically valuable crops such as tomato, beans, and rice (2, 3). *P. syringae* pv. tomato strain DC3000, which infects tomato crops, as well as the model plant *Arabidopsis thaliana*, has been fundamental in increasing our understanding of *P. syringae* pathogenicity. Found in seeds, soil, rotting plant material, and on leaf surfaces (2, 4), *P. syringae* pv. tomato DC3000 enters the plant through wounds or leaf stomata and then replicates within the apoplast, eventually causing chlorosis (yellowing), necrotic lesions, and programmed cell death in incompatible interactions (2, 5, 6).

As with many other Gram-negative plant and animal pathogens, the virulence of *P. syringae* relies upon a type III secretion system (T3SS)—a needlelike appendage that facilitates the delivery of virulence effectors into the host cells (5, 7). The T3SS of *P. syringae* is encoded by the hypersensitive response (HR) and pathogenicity (*hrp*) and HR and conserved (*hrc*) gene cluster (5) that is controlled by the extracytoplasmic function sigma factor HrpL (8). The expression of HrpL is strictly controlled by sigma-54 and cooperatively activated through the enhancer binding proteins HrpRS (8, 9). Transcriptional control through HrpL and HrpRS is not limited to the *hrp-hrc* T3SS cluster but extends to other genes, including some which have unknown roles in *P. syringae* pathogenicity (10). One of these genes is PSPTO_2907, otherwise known as *chp8* (co-regulated with *hrp* *8*) (10), whose role in pathogenicity we have investigated in this study.

## RESULTS

### Chp8 is embedded in the Hrp regulon, and its expression is activated by plant signals.

**FIG 1:**
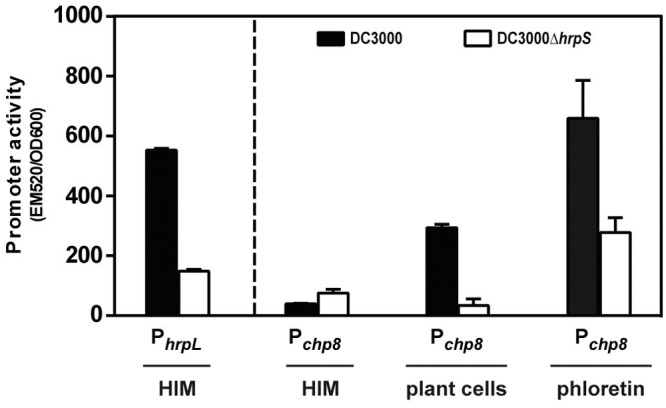
Activity of the *chp8* promoter. The activity of the *chp8* promoter was measured in *P*. *syringae* pv. tomato DC3000 and DC3000Δ*hrpS* in *hrp*-inducing medium (HIM) in the presence of plant cells or the plant flavonoid phloretin. Promoter activity was reported via production of GFP and expressed as the ratio of fluorescence intensity at 520 nm and OD_600_. P_*hrpL*_, cells contain a reporter fusion of the *hrpL* promoter to *gfp*; P_*chp8*_, cells contain a reporter fusion of the *chp8* promoter to *gfp*. Error bars show standard errors of the means. Statistical analysis of P*_chp8_* activity using unpaired *t* test gave results as follows (significant if *P* value is <0.05): DC3000 (HIM) versus DC3000Δ*hrpS* (HIM) was not significant, *P* = 0.0544; DC3000 (HIM) versus DC3000 (plant cells) was significant, *P* < 0.0001; DC3000 (plant cells) versus DC3000Δ*hrpS* (plant cells) was significant, *P* = 0.0005; DC3000 (HIM) versus DC3000 (phloretin) was significant, *P* = 0.0078; DC3000 (phloretin) versus DC3000Δ*hrpS* (phloretin) was significant, *P* = 0.0478.

A functional genomics analysis of *P. syringae* pv. tomato strain DC3000 identified *chp8* as a novel Hrp-regulated gene whose expression was upregulated under Hrp-inducing conditions, apparently in a *hrpRS*-dependent but *hrpL*-independent manner (10, 11). To confirm these findings, we measured the activity of the *chp8* promoter in strain DC3000 in the presence and absence of *hrpS* (Fig. 1) or *hrpL* (see Fig. S1 in the supplemental material), respectively. Initially, we measured Chp8 induction in HIM (*hrp*-inducing medium), since it has been shown to induce *hrp-hrc* gene expression (Fig. 1, P_*hrpL*_), presumably by mimicking the nutritionally depleted environment encountered by DC3000 in the apoplast (12, 13). However, we could not detect upregulation of *chp8* induction in DC3000 in HIM alone (Fig. 1, P_*chp8*_ HIM). We reasoned that *chp8* induction may, in addition, require plant-derived signals. Indeed, the activity of the *chp8* promoter was markedly increased when DC3000 was grown in a plant cell culture (Fig. 1, P_*chp8*_ plant cells). Recent studies have identified that plants produce flavonoids upon infection with *P. syringae* pv. tomato DC3000 and that this pathogen is susceptible to the plant flavonoid phloretin (14). To determine whether phloretin affects *chp8* induction, we measured the activity of the *chp8* promoter in HIM supplemented with phloretin (Fig. 1, P_*chp8*_ phloretin). As shown by the results in Fig. 1, the activity of the *chp8* promoter was markedly increased in the presence of phloretin. In line with the requirement of HrpRS for *chp8* induction, the positive effect of plant cells and phloretin is diminished in the absence of *hrpS* (Fig. 1, DC3000Δ*hrpS*). Extending earlier observations (10, 11), these results demonstrate that Chp8 is indeed embedded in the Hrp regulon, suggesting that coregulation of Chp8 and T3SS occurs and that induction is responsive to plant signals, implying a role in the infection process.

### Chp8 exhibits a functional c-di-GMP synthase activity *in vivo* and promotes a sessile lifestyle of *P*. *syringae* pv. tomato DC3000.

**FIG 2:**
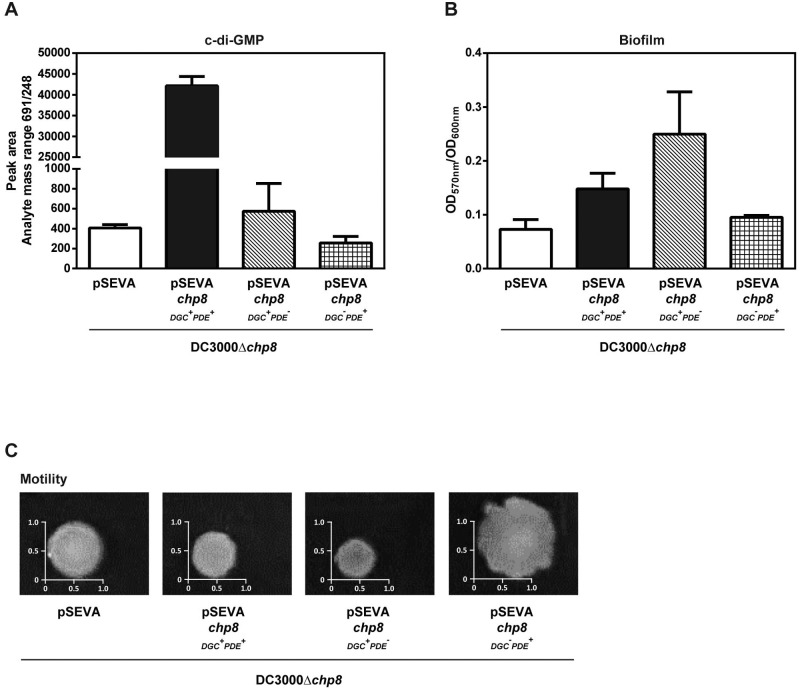
Effects of Chp8 on c-di-GMP production, biofilm formation, and motility of *P*. *syringae* pv. tomato DC3000 strains. (A) Cellular c-di-GMP levels were analyzed by LC-MS/MS. Shown are the peak area data from the c-di-GMP-specific analyte mass range 691/248. Error bars show standard errors of the means. Statistical analysis using unpaired *t* test gave results as follows (significant if *P* value is <0.05): DC3000Δ*chp8*/pSEVA versus DC3000Δ*chp8*/pSEVA*chp8*_DGC_^+^_PDE_^+^ was significant, *P* = 0.0029; DC3000Δ*chp8*/pSEVA*chp8*_DGC_^+^_PDE_^+^ versus DC3000Δ*chp8*/pSEVA*chp8*_DGC_^+^_PDE_^−^ was significant, *P* = 0.003; DC3000Δ*chp8*/pSEVA*chp8*_DGC_^+^_PDE_^+^ versus DC3000Δ*chp8*/pSEVA*chp8*_DGC_^−^_PDE_^+^ was significant, *P* = 0.0029; DC3000Δ*chp8*/pSEVA*chp8*_DGC_^+^_PDE_^−^ versus DC3000Δ*chp8*/pSEVA*chp8*_DGC_^−^_PDE_^+^ was not significant, *P* = 0.3856. (B) Biofilm formation was measured by the crystal violet-staining method and expressed as the ratio of the optical densities at 570 nm and 600 nm. Error bars show standard errors of the means. Statistical analysis using unpaired *t* test gave results as follows (significant if *P* value is <0.05): DC3000Δ*chp8*/pSEVA versus DC3000Δ*chp8*/pSEVA*chp8*_DGC_^+^_PDE_^+^ was significant, *P* = 0.0192; DC3000Δ*chp8*/pSEVA*chp8*_DGC_^+^_PDE_^+^ versus DC3000Δ*chp8*/pSEVA*chp8*_DGC_^+^_PDE_^−^ was not significant, *P* = 0.1037; DC3000Δ*chp8*/pSEVA*chp8*_DGC_^+^_PDE_^+^ versus DC3000Δ*chp8*/pSEVA*chp8*_DGC_^−^_PDE_^+^ was significant, *P* = 0.0352. (C) Motility was measured as the diameter of bacterial spread on soft (0.4%) agar plates. DC3000Δ*chp8*/pSEVA, vector control; DC3000Δ*chp8*/pSEVA*chp8*_DGC_^+^_PDE_^+^, cells expressing wild-type Chp8; DC3000Δ*chp8*/pSEVA*chp8*_DGC_^+^_PDE_^−^, cells expressing Chp8 with intact GGDEF and inactivated EAL domain; DC3000Δ*chp8*/pSEVA*chp8*_DGC_^−^_PDE_^+^, cells expressing Chp8 with intact EAL and inactivated GGDEF domain.

*In silico* analyses of Chp8 (see Fig. S2 in the supplemental material) infer that it belongs to the diguanylate cyclase (DGC) and/or the phosphodiesterase (PDE) family of proteins. The presence of a GGDEF (characteristic of a DGC) and an EAL (characteristic of a PDE) domain indicates that Chp8 has active cyclic di-GMP (c-di-GMP)-synthesizing (DGC) and/or -degrading (PDE) activities (15–17). As a second messenger, c-di-GMP often controls the switch between planktonic and sessile lifestyles (15–17). DGCs, as c-di-GMP producers, promote biofilm formation and decrease motility, while PDEs, as c-di-GMP degraders, promote motility and decrease biofilm formation (15–17). To determine which of the two opposing activities of Chp8 predominates *in vivo*, we measured the (i) cellular c-di-GMP levels, (ii) biofilm formation, and (iii) motility of *P. syringae* pv. tomato DC3000 in the presence and absence (Fig. S3) and upon ectopic expression of wild-type Chp8 and two Chp8 variants with either the DGC or the PDE domain inactivated (Fig. 2). We used ectopic expression instead of phloretin-induced Chp8 expression since phloretin had such a strong effect on the phenotypes tested that it masked any Chp8-specific changes. Consistent with our earlier observations that *chp8* induction required plant-derived signals, heterologous ectopic expression was needed to study Chp8 function *ex planta* (Fig. 2 and Fig. S3). Strikingly, cells expressing wild-type *chp8* (*P. syringae* pv. tomato DC3000Δ*chp8*/pSEVA*chp8*_DGC_^+^_PDE_^+^) showed a marked increase in cellular c-di-GMP (Fig. 2A), a slightly more extensive biofilm (Fig. 2B), and decreased motility (Fig. 2C), in line with net c-di-GMP production by Chp8 *in vivo*.

To test the activities of the DGC and PDE domains of Chp8 independently, we replaced the critical signature amino acids GGDEF and EAL (see Fig. S2 in the supplemental material) with alanine to create Chp8_DGC_^−^_PDE_^+^ (disrupting the GGDEF but maintaining the integrity of the EAL motif) and, conversely, Chp8_DGC_^+^_PDE_^−^ (disrupting the EAL but maintaining the integrity of the GGDEF motif). Mutating the Chp8 GGDEF motif (*P. syringae* pv. tomato DC3000Δ*chp8*/pSEVA*chp8*_DGC_^−^_PDE_^+^) impairs c-di-GMP production and biofilm formation (Fig. 2A), demonstrating that Chp8 indeed encodes a functional DGC domain. Interestingly, the Chp8 PDE domain appears to be functional (*P. syringae* pv. tomato DC3000Δ*chp8*/pSEVA*chp8*_DGC_^−^_PDE_^+^), causing a marked increase in the motility of the cells compared to that of the vector control (Fig. 2C). Remarkably, inactivation of the Chp8 PDE domain (*P. syringae* pv. tomato DC3000Δ*chp8*/pSEVA*chp8*_DGC_^+^_PDE_^−^) also interferes with c-di-GMP production (Fig. 2A) despite an unmodified DGC domain, indicating that both domains are required for maximal c-di-GMP synthase activity of Chp8. However, the DGC domain of Chp8 alone, in the absence of the intact PDE domain, retains its characteristic phenotypic impact, evident through an extensive biofilm (Fig. 2B) and decreased motility (Fig. 2C). In summary, we conclude that Chp8 is a composite diguanylate cyclase in which both the DGC and PDE domains are active and required for maximal c-di-GMP synthase activity *in vivo* and that Chp8 is involved in the switch toward a sessile lifestyle of *P. syringae* pv. tomato DC3000 by promoting biofilm formation and decreasing motility.

### Chp8 downregulates flagellin and upregulates EPS production of *P*. *syringae* pv. tomato DC3000.

**FIG 3:**
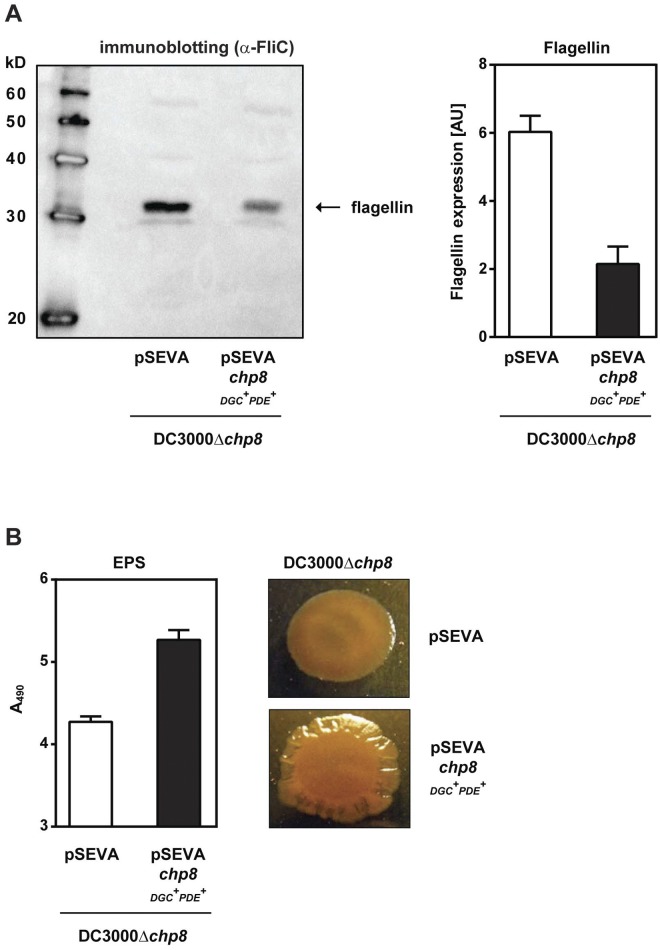
Effects of Chp8 on flagellin and EPS production in *P*. *syringae* pv. tomato DC3000 strains. (A) The effect of Chp8 on flagellin production was measured via immunoblotting with antibodies against FliC (77). The band corresponding to flagellin was quantified via densitometry, taking into account gel loading. The results for the loading control can be found in Fig. S4 in the supplemental material. Statistical analysis using unpaired *t* test gave results as follows (significant if *P* value is <0.05): DC3000Δ*chp8*/pSEVA versus DC3000Δ*chp8*/pSEVA*chp8*_DGC_^+^_PDE_^+^ was significant, *P* = 0.0310. AU, arbitrary units. (B) The effect of Chp8 on EPS production was measured via the change in absorbance at 490 nm through retention of the Congo red cell stain and visualized through the formation of wrinkly colony morphology. Statistical analysis using unpaired *t* test gave results as follows (significant if *P* value is <0.05): DC3000Δ*chp8*/pSEVA versus DC3000Δ*chp8*/pSEVA*chp8*_DGC_^+^_PDE_^+^ was significant, *P* = 0.0004. DC3000Δ*chp8*/pSEVA, vector control; DC3000Δ*chp8*/pSEVA*chp8*_DGC_^+^_PDE_^+^, cells expressing wild-type Chp8. Error bars show standard errors of the means.

The Chp8-dependent changes in motility prompted us to investigate the impact of Chp8 on flagellin production. Flagellin is the principal constituent of bacterial flagella, which confer bacterial motility (18). Flagellin is also one key pathogen-associated molecular pattern (PAMP) used by plants to detect the presence of a pathogen (19–23). Central to pathogenicity, therefore, is the link between pathogen detection and plant disease resistance via changes in the phytohormone homeostasis (24–26). Once detected by the PAMP system, flagellin results in the accumulation of the phytohormone salicylic acid (SA) and downstream SA-dependent defense responses in the plant (21–23). Consequently, *Arabidopsis* plants that are unable to detect flagellin exhibit more severe disease symptoms and are less resilient to infection (22). Interestingly, we found that the flagellin levels decreased significantly upon the expression of Chp8 in *P. syringae* pv. tomato DC3000Δ*chp8* (Fig. 3A, pSEVA*chp8*_DGC_^+^_PDE_^+^). The data are fully in line with our phenotypic observations of a Chp8-dependent decrease in the motility of strain DC3000 (Fig. 2) and point toward a role for Chp8 in undermining the SA-dependent plant immune system.

The detection of PAMPs, such as flagellin, generates a cytosolic influx of Ca^2+^ into the plant cell (27). Here, Ca^2+^ acts as a second messenger modulating SA biosynthesis and SA-dependent immune responses (28). Bacteria, in turn, chelate Ca^2+^, suppressing PAMP-triggered plant immunity through the production of polyanionic extracellular polysaccharides (EPS) (29). Notably, EPS production is c-di-GMP dependent and is thus interlinked with DGC action (30). Chp8’s DGC activity (Fig. 2) prompted us to assess the impact of Chp8 on EPS production. We utilized the observation that EPS increases the cell’s ability to retain Congo red and to form a “wrinkly” colony (31). As shown by the results in Fig. 3B, cells expressing Chp8 (Fig. 3B, pSEVA*chp8*_DGC_^+^_PDE_^+^) retained more Congo red and were markedly more wrinkly in colony morphology than cells lacking Chp8 (Fig. 3B, pSEVA). Taken together, the data show that Chp8 downregulates flagellin and increases EPS production. Chp8 may therefore hinder the detection of *P. syringae* pv. tomato DC3000 by the plant and so help to circumvent PAMP-triggered immunity and promote DC3000’s pathogenicity.

### Chp8 promotes *P*. *syringae* pv. tomato DC3000’s pathogenicity.

Our data show that Chp8 is embedded in the same regulon as the T3SS and that its expression is induced by plant signals and causes a decrease in flagellin and an increase in EPS production. Together, these results strongly indicate a role for Chp8 in the pathogenesis of a *P. syringae* pv. tomato DC3000 infection. To test this proposal, we infected *Arabidopsis thaliana* plants with strain DC3000 or the DC3000Δ*chp8* mutant using a plate-flooding technique (32) and, in each case, monitored plant health postinfection. *P. syringae* pv. tomato DC3000 relies on motility to enter the apoplast of the host plant through openings on the surface of the leaves (e.g., stomata), and thus, the infectivity of immotile cells is markedly reduced (33–36). Since Chp8 decreases the motility of *P. syringae* pv. tomato DC3000, we chose to flood the plants with low-titer bacterial suspensions as an alternative to leaf wounding or infiltration methods that permit passive entry, in order to encourage an infection route that requires an active movement of the bacterial cells into the apoplast through the stomata.

**FIG 4:**
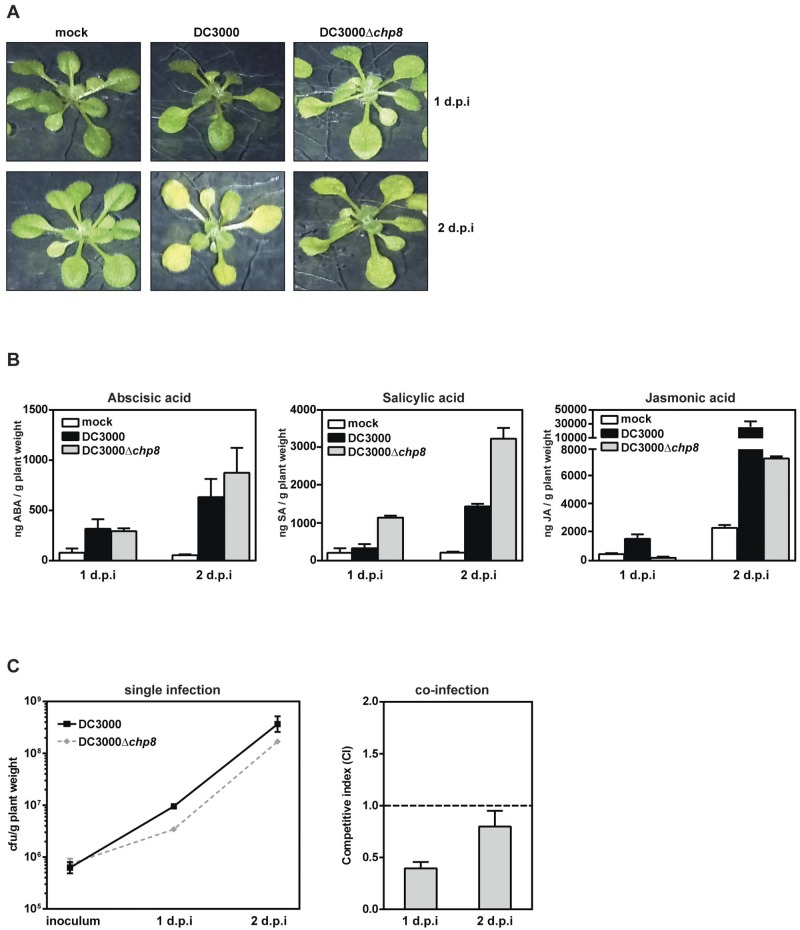
Effects of Chp8 on *P*. *syringae* pv. tomato DC3000 apoplast colonization and disease symptom development and hormonal immune responses of the plant. (A) Disease symptom development (yellowing of leaves) was followed after single infection of *Arabidopsis thaliana* with either DC3000 or DC3000Δ*chp8*. Mock treatment was included as a negative control. Shown are representative images taken 1 and 2 d.p.i. (B) Levels of abscisic acid (ABA), salicylic acid (SA), and jasmonic acid (JA) were measured after single infection of *Arabidopsis thaliana* with either DC3000 or DC3000Δ*chp8*. Mock treatment was included as a negative control. Shown are the levels measured 1 and 2 d.p.i. Statistical analysis using unpaired *t* test gave results as follows (significant if *P* value is <0.05): ABA at 1 d.p.i., DC3000 versus DC3000Δ*chp8* was not significant, *P* = 0.8307; ABA at 2 d.p.i., DC3000 versus DC3000Δ*chp8* was not significant, *P* = 0.5139; SA at 1 d.p.i., DC3000 versus DC3000Δ*chp8* was significant, *P* = 0.011; SA at 2 d.p.i., DC3000 versus DC3000Δ*chp8* was significant, *P* = 0.0134; JA at 1 d.p.i., DC3000 versus DC3000Δ*chp8* was significant, *P* = 0.0458; JA at 2 d.p.i., DC3000 versus DC3000Δ*chp8* was not significant, *P* = 0.144. (C) Chp8-dependent differences in apoplast colonization were assessed 1 and 2 d.p.i. by measuring CFU/g plant weight (left panel) after single infection with either DC3000 or DC3000Δ*chp8* and by calculating the competitive index (CI) after coinfection with both strains at a 1:1 ratio. For CI, the numerator is CFU/g plant recovered from the apoplast (DC3000Δ*chp8*/DC3000) and the denominator is CFU in the initial inoculum (DC3000Δ*chp8*/DC3000), and values indicate results as follows: CI < 1, mutant is less competitive than wild-type; CI = 1, mutant and wild-type are equally competitive; CI > 1, mutant is more competitive than wild-type. Statistical analysis using linear regression for single infection and unpaired *t* test for coinfection gave results as follows (significant if *P* value is <0.05): single infection, inoculum to 1 d.p.i., DC3000 (*y* = 8.9e^6^ × −625,000, *R*^2^ = 0.9763) versus DC3000Δ*chp8* (*y* = 2.69e^6^ × −735,000, *R*^2^ = 0.9681) was significant, *P* = 0.0039; single infection, 1 d.p.i. to 2 d.p.i., DC3000 (*y* = 3.57e^8^ × −3.474e^8^, *R*^2^ = 0.7377) versus DC3000Δ*chp8* (*y* = 1.656e^8^ × −1.622e^8^, *R*^2^ = 0.9974) was not significant, *P* = 0.2727; coinfection (CI ≠ 1), 1 d.p.i. was significant, *P* = 0.0048, and 2 d.p.i. was not significant, *P* = 0.1983. Error bars show standard errors of the means.

One characteristic symptom of *P. syringae* pv. tomato DC3000 infection is yellowing (chlorosis) of leaves (2, 6). Both strains elicited chlorosis of *Arabidopsis thaliana* and ultimately caused plant death. However, the prevalence of disease symptoms was markedly delayed upon infection with the DC3000Δ*chp8* mutant compared to the results with DC3000 (Fig. 4A), indicating that Chp8 negatively affects the resilience of the plants.

To elucidate the molecular basis of the Chp8-dependent differences in disease progression, we quantified the levels of three key hormones, abscisic acid (ABA), salicylic acid (SA), and jasmonic acid (JA), employed by the plant to modulate its immune response against infection (24, 37). ABA regulates plant development in response to abiotic stresses (38, 39); it also increases the plant’s susceptibility to pathogens, and thus, *P. syringae* pv. tomato DC3000 employs T3SS effectors during infection to increase ABA (40, 41). Accordingly, we observed a marked increase in ABA levels upon infection with DC3000 (Fig. 4B). However, given that similar ABA levels were observed upon infection with DC3000Δ*chp8* (Fig. 4B), it seems that the effect Chp8 has on pathogenicity is not associated with the ABA system.

As described above, plants respond to an attack by (hemi-)biotrophic pathogens like *P. syringae* pv. tomato DC3000 by accumulating SA (42, 43) and to herbivores and necrotrophic pathogens by accumulating JA (44). High levels of either SA or JA, which are regulated antagonistically (42), trigger a range of plant immune responses to combat the infection (45–48). Unsurprisingly, SA accumulated upon infection with both strains (Fig. 4B). Strikingly, however, since larger quantities of SA were recovered from plants infected with DC3000Δ*chp8*, it would appear that Chp8 restricts SA accumulation (Fig. 4B and see Fig. S5 in the supplemental material). This is fully in line with our observations that Chp8 decreases flagellin and increases EPS production, which are known to affect SA levels (Fig. 3) (21–23, 29). Cross-talk between SA and JA has been demonstrated (42, 44). During systemic acquired resistance, an initial wave of JA signaling precedes a wave of SA signaling (49), and JA levels then decrease (44, 49, 50). In agreement with this antagonism between SA and JA (42), we found that JA levels were higher in plants infected with DC3000 (Fig. 4C and Fig. S5) than in those infected with the DC3000Δ*chp8* mutant. Moreover, we conclude that Chp8 decreased JA indirectly through elevated SA levels. Elevated SA would result in negative regulation of JA and explain the observations reported here.

To investigate at what stage during the infection Chp8 is particularly important, we compared apoplast colonization after single and coinfections with *P. syringae* pv. tomato DC3000 and DC3000Δ*chp8* (Fig. 4C). Notably, after stimulating infections with single strains, we recovered significantly more DC3000 cells than DC3000Δ*chp8* cells from plants 1 day postinfection (d.p.i.), despite similar initial inoculum densities (Fig. 4C). However, between day 1 and day 2 postinfection, the bacterial load increased similarly for both strains (Fig. 4C). Consistent with this outcome, after a 1:1 coinfection with both strains, the competitive index for DC3000Δ*chp8* on day 1 postinfection was only ~0.4 (standard error of the mean [SEM], 0.06), but it increased to ~0.8 (SEM, 0.15) on day 2 postinfection (Fig. 4C). Apparently, apoplast colonization during early infection events is impaired in DC3000Δ*chp8* cells. Chp8, however, appears to have no effect on the survival of DC3000 within the apoplast at later stages of infection (Fig. 4C).

Taken together, our plant infection studies showed that Chp8 specifically affected the SA/JA hormone levels, ultimately affecting *P. syringae* pv. tomato DC3000 pathogenesis in a manner that was particularly apparent in the early stages of infection.

## DISCUSSION

Plant-pathogen interactions are characterized by the sophisticated interplay between plant immunity elicited upon pathogen recognition, via PAMPs, and immune evasion by the pathogen (51–53). One of the key PAMPs through which plants, and indeed other hosts, recognize pathogens is the structural component of bacterial flagella, flagellin, and specifically, the flg22 epitope (19–23). Recognition of flagellin occurs during both epi- and endophytic growth of *P. syringae*, triggering Ca^2+^ influx into plant cells (28) and SA-dependent defense mechanisms, such as stomatal closure (48), induction of pathogenesis-related (PR) antimicrobial proteins (46), increased reactive oxygen species (45), and enhanced callose deposition (47). However, since flagellar motility enhances epiphytic fitness and enables bacteria to actively enter the apoplast (33–36), pathogens have evolved strategies to diminish flagellin-dependent detection by the plant immune system (19–23). For instance, some *Xanthomonas campestris* strains evade detection due to polymorphisms of the flg22 epitope (54). *P. syringae* suppresses flagellin-triggered immunity by reducing the expression of flagellar genes at both the transcriptional (11) and translational level (55) and by blocking formation of the FLS2-BAK_1_ flagellin receptor cluster of the plant (56). Our studies now show that Chp8 also contributes to the major effort of *P. syringae* pv. tomato DC3000 to diminish PAMP-triggered plant immune responses.

Extending the results of previous reports (10, 11), we show that Chp8 is embedded in the Hrp regulon in a way that suggests that signal transduction downstream from HrpRS bifurcates into HrpL-dependent (T3SS) and HrpL-independent (e.g., Chp8) pathways. Bifurcation thereby appears to occur in response to nutritional (HrpL-dependent pathway) (12) or plant-derived signals (HrpL-independent pathway).

Chp8 is a composite GGDEF-EAL protein. Despite the overriding activity of the Chp8 DGC domain, its EAL domain retained PDE activity (illustrated by the increased motility of the Chp8_DGC_^−^_PDE_^+^ variant) but, more importantly, appeared to functionally interact with the DGC for maximal c-di-GMP production. Similar functional requirements for full DGC and/or PDE activities have also been reported for the composite GGDEF-EAL proteins FimX from *Pseudomonas aeruginosa* (57) and MSDGC-1 from *Mycobacterium smegmatis* (58). In addition to the GGDEF motif in the active (A) site, many DGCs also contain a secondary inhibitory (I) site to regulate c-di-GMP production through feedback inhibition (15, 16) (see Fig. S2 in the supplemental material). Recent *in silico* analyses point to a correlation between I site conservation and the presence of an EAL domain, where 66% of GGDEF-only and 48% of composite GGDEF-EAL proteins contain intact I and A sites (59). The EAL domain may therefore compensate for loss of the I site by directly regulating the output of the GGDEF domain. Since Chp8 also lacks an intact I site (due to replacement of the RXXD motif with an SXXV motif) (Fig. S2), this may explain the cooperative effect of the EAL domain. Our observations that the biofilm and motility phenotypes of wild-type Chp8 resemble an intermediate state between the phenotypes of the Chp8_DGC_^+^_PDE_^−^ and Chp8_DGC_^−^_PDE_^+^ variants further supports this notion. Interestingly, the role of c-di-GMP in virulence is consistent with the proposed subdivision of the DGC and PDE pool of a bacterial cell to specific physiological processes (60–63). Hence, this may be why c-di-GMP can negatively affect the virulence of the plant pathogens *Xanthomonas campestris* (64, 65) and *Erwinia amylovora* (66) and yet positively (by means of Chp8) affect *P. syringae* pv. tomato DC3000 virulence and disease progression.

The consequences of Chp8 expression are increased c-di-GMP production, decreased flagellin production (and by association, motility), and increased EPS production—ultimately escalating the susceptibility of *Arabidopsis thaliana* to infection. The mechanism underpinning Chp8-dependent *P. syringae* pv. tomato DC3000 pathogenicity, i.e., c-di-GMP production resulting in evasion of flagellin-triggered plant immune responses, seems generalizable. Strikingly, the accumulation of interleukin-8, a proinflammatory cytokine involved in innate immunity, is impaired by high c-di-GMP levels upon *Salmonella enterica* serovar Typhimurium infection of HAT-29 cells (67) and is flagellin triggered in intestinal epithelial cells upon enteroaggregative *Escherichia coli* infection (68).

Our findings that *chp8* expression is responsive to plant signals are supported through recent transcriptional studies which showed that the expression of Psyr_2711, the *chp8* homologue in *P. syringae* pv. syringae B728a, increased during epiphytic growth (69). These data further indicate that Chp8 acts prior to the passage of *P. syringae* pv. tomato DC3000 through the stomata (in line with reports that phloretin, which we show induces Chp8 expression, is a cuticular flavonoid [70]) during the early stages of infection. Recall that, compared to the results for DC3000, the onset of disease symptoms and apoplast colonization by DC3000Δ*chp8* are reduced on day 1 but similar in later stages of infection.

In summary, among the host-pathogen interactions that depend upon complex interplays between pathogen-triggered host immunity and pathogen evasion of the host immune response, Chp8, a composite GGDEF-EAL protein with a net c-di-GMP activity, serves to reduce flagellin production and increase EPS production, thus functioning as a contributor to pathogen survival in this finely tuned balancing act.

## MATERIALS AND METHODS

### Bacterial strains and growth conditions.

Unless otherwise indicated, *P. syringae* pv. tomato DC3000 and its derivatives were grown at 28°C in King’s B (KB) medium or Hrp-inducing minimal medium (HIM) supplemented with 50 µg/ml rifampin and additional antibiotics as appropriate (50 µg/ml kanamycin and 100 µg/ml ampicillin).

### Construction of gene deletions in *P*. *syringae* pv. tomato DC3000.

Markerless *P. syringae* pv. tomato strains DC3000Δ*chp8*, DC3000Δ*hrpS*, and DC3000Δ*hrpL* were constructed via allelic exchange, utilizing a protocol adapted from reference 71. Briefly, ~2- to 700-bp sequences, corresponding to the 5′ and 3′ flanking regions of the target open reading frame (ORF), were PCR amplified and fused by single overlap extension PCR. The fusions were inserted into pGEM-T (Promega) to generate intermediate pFUSE vectors. A BamHI fragment containing an FRT-flanked kanamycin resistance gene (*nptII*) was obtained from the pGEM-T-*nptII*-BamHI plasmid and inserted into the pFUSE constructs to yield pKO plasmids. *P. syringae* pv. tomato DC3000 was electroporated with pKOchp8, pKOhrpS, and pKOhrpL. Recombinants were selected on LB-kanamycin and screened for ampicillin sensitivity to distinguish allelic exchange (a double recombination event) from whole-plasmid integration (single recombination). To avoid polar transcriptional effects due to the *nptII* promoter, the *nptII* cassette was excised from the resulting *Δchp8* ∷*nptII*, *ΔhrpS* ∷*nptII*, and *ΔhrpL* ∷*nptII* strains via recombination at FRT sites flanking the *nptII* gene through the expression of FLP recombinase from the pFLP2 plasmid (72). Transformants were screened for loss of kanamycin resistance. Cured mutant strains carrying pFLP2 were subcultured four times in LB medium supplemented with 5% sucrose for *sacB*-mediated counterselection of the plasmid. Single colonies were scored for ampicillin sensitivity as an indication of plasmid loss. Deletion of *chp8*, *hrpL*, and *hrpS* was confirmed by sequencing. All primers used are listed in Table S1 in the supplemental material.

### Construction of plasmids.

To create the green fluorescent protein (GFP) gene-tagged reporter for *chp8* promoter activity, a 648-bp fragment upstream from the PSPTO_2907 start site was cloned into pBBR1MCS-4 (73) containing *gfp-mut3*, including the *rbs30* ribosome binding site and transcriptional terminator (74). For ectopic expression, Chp8 was placed under the heterologous control of a *lacI*^q^-P_*trc*_ module by cloning the PSPTO_2907 coding sequence into pSEVA224 (75) to create pSEVA*chp8*_DGC_^+^_PDE_^+^. This vector also contains an RK2 origin of replication, conferring broad host range and low copy number. The GGDEF and EAL motifs of Chp8 were replaced with alanine residues via site-directed mutagenesis to specifically inactivate the DGC and PDE domains, respectively, leaving the rest of the protein intact. Plasmid pSEVA*chp8*_DGC_^−^_PDE_^+^ encodes Chp8_GGDEF::AAAAA_, while pSEVA*chp8*_DGC_^+^_PDE_^−^ encodes Chp8_EAL::AAA_. Sequencing confirmed successful construction of the plasmids. All primers and plasmids used are listed in Table S1.

### Measurement of *chp8* promoter activity *ex planta.*

Overnight cultures of *P. syringae* pv. tomato DC3000 strains carrying the pBBR1-P_*chp8*_-*gfp* reporter were washed twice with 10 mM MgCl_2_ and resuspended in HIM medium with 10 mM fructose to an optical density at 600 nm (OD_600_) of 0.25. To test the effect of phloretin, cell cultures were supplemented with 1 mM of the flavonoid. Fluorescence was measured simultaneously with cell density (OD_600_) in a black, clear-bottom 96-well tissue culture plate using a BMG FLUOstar fluorometer (485 nm for excitation, 520 ± 10 nm for emission, gain of 1,000). The fluorescence per unit of cell growth was calculated in triplicate at 20-min intervals over 8 h. Growth was at 25°C with orbital shaking at 200 rpm.

### Measurement of *chp8* promoter activity in plant cell coculture.

A suspension of *Arabidopsis thaliana* (Landsberg erecta) callous cells was kindly provided by Alessandra Dovoto (Royal Holloway University, London). The suspension was maintained in a 16-h light regimen at 20°C and subcultured 10-fold every 7 days into cell suspension medium (3% sucrose, 0.44% MSMO (Murashige & Skoog medium with minimal organics), 2.7 µM 1-naphthylacetic acid, 50 µg/liter kinetin solution, pH 5.7). Ten milliliters of plant cell suspension was harvested during stationary-phase growth (approximately 2 to 3 days into the 7-day culture cycle) for use in coculture experiments. The cell suspension medium was replaced with HIM, with or without *P. syringae* pv. tomato DC3000 cells containing the pBBR-P_*chp8*_-*gfp* reporter (prepared as described above), during a series of three cycles in which the plant cells were allowed to sediment, after which the medium layer was aspirated. The activity of the *chp8* promoter was measured using the fluorescent reporter assay as described above, except that the supplementation of HIM with a 10 mM carbon source was omitted.

### Measurement of cellular c-di-GMP.

For c-di-GMP measurement, bacterial cells were grown on King’s B solid agar plates overnight and resuspended in King’s B growth medium at an OD_600_ of 1. The suspension was supplemented with 200 ng/ml cyclic XMP (cXMP) as an internal standard. Extraction of c-di-GMP and cXMP was done in acetonitrile-methanol-water (40:40:20, vol/vol/vol) as described previously (76). c-di-GMP analysis was by liquid chromatography–tandem mass spectrometry (LC-MS/MS) (76, 77). The LC-MS system was comprised of an Agilent 1100 LC system and an ABSciex 6500 Qtrap MS. c-di-GMP was separated on a Phenomenex Luna C_18_(2) column (100 mm by 2 mm by 3 µm) at a temperature of 35°C, utilizing a gradient solvent system comprised of solvents A (10-mM ammonium acetate and 0.1% [vol/vol] formic acid) and B (acetonitrile). The compounds were eluted at a flow rate of 400 ml/min with a gradient from 100% A to 90% A over 5 min. The column was washed with 70% B for 3 min and re-equilibrated with 100% A. Typically, 20-µl injections were used for the analysis. The MS was configured with a Turbo Spray IonDrive source; gas 1 and 2 were set to 40 and 60, respectively; the source temperature was 425°C; and the ion spray voltage was 5,500 V. c-di-GMP and cXMP were analyzed by multiple-reaction monitoring (MRM) in positive mode using the following transitions (mass-to-charge ratio [*m*/*z*]), with the collision energies (CE) used shown in parentheses after the transitions: 691.1→152 (60 eV), 691.1→248 (50 eV), 691.1→540 (40 eV), 347→153 (30 eV), and 347→136 (60 eV). The declustering potential, exit potential, and collision cell exit potential were set at 80 V, 10 V, and 10 V, respectively, for all transitions. c-di-GMP and cXMP eluted with retention times of 4.6 min and 5.0 min, respectively. Data acquisition and analysis were done with Analyst 1.6.1.

### Biofilm formation.

Biofilm formation was assayed as described previously (78). Briefly, *P. syringae* pv. tomato DC3000 strains were grown in KB in borosilicate glass tubes for 48 h at 28°C without shaking. Planktonic cells were removed, and the OD_600_ was measured. Sessile cells bound to the glass were washed twice with water, dried, and stained through a 15-min incubation with 0.1% crystal violet (Sigma) at room temperature. After washing twice with water, the stained cells were resuspended in 75% ethanol and the OD_590_ was measured. Biofilm formation was expressed as the ratio between sessile and planktonic cells (OD_590_/OD_600_). All assays were performed in triplicate.

### Motility assay.

For motility, cells from overnight cultures were diluted in fresh medium to an OD_600_ of 0.05. Five microliters of the bacterial suspension were spotted onto soft agar plates containing 0.4% agar. The plates were incubated at 28°C for 48 h, and swarming across the plate was measured as the diameter of spread. All assays were performed in triplicate.

### Flagellin quantification.

Flagellin production was measured via immunoblotting with antibodies against FliC as described previously (79). Briefly, bacteria were pelleted by centrifugation at 5,000 rpm for 10 min. To extract flagellin, pellets were washed and resuspended in 50 mM Tris-HCl, pH 8.0, 150 mM NaCl, 0.1% Tween 20 (vol/vol), 10% glycerol (vol/vol), 1 mM phenylmethylsulfonyl fluoride. Cells were sonicated, and proteins precipitated with 5% trichloroacetic acid for 1 h on ice, resuspended in the same buffer, and separated by 12% SDS-PAGE. Flagellin was detected via immunoblotting with anti-FliC antibodies (79) and quantified via densitometry. Gel loading was controlled and quantified via SYPRO ruby protein stain (Molecular Probes).

### EPS quantification and colony morphology.

Extracellular polysaccharide (EPS) was quantified and colony morphology visualized as described previously (30). Briefly, overnight cultures of *P. syringae* pv. tomato DC3000Δ*chp8*/pSEVA and DC3000Δ*chp8*/pSEVA*chp8*, respectively, were washed with fresh medium, 40 µg/ml Congo red cell stain (Alfa Aesar) was added, and the bacterial suspension was incubated at room temperature for 2 h with shaking. Cells were pelleted, washed, and resuspended in fresh medium. Cells were normalized by protein content, and the absorbance at 490 nm was measured to quantify the amount of Congo red retained by the cells. To visualize colony morphology, cells from overnight cultures were diluted in fresh medium to an OD_600_ of 0.05. Five-microliter amounts of the bacterial suspensions were spotted onto solid KB agar plates and incubated at 28°C for 5 days before visualizing colony morphology. All assays were performed in triplicate.

### Plant infection assays.

Arabidopsis Col-0 seedlings were grown on agar plates composed of 1/2 strength (2.1 g/liter) MS (Murashige & Skoog) medium, 0.546 g/liter MES [2-(*N*-morpholino)ethanesulfonic acid], 1% sucrose, and 1% phytagel. Seeds were vernalized for 2 days at 4°C prior to sterilization. Seeds were sterilized as follows: 5 min of 70% ethanol, 5 min of 50% sodium hypochloride, and 4 washes with sterile distilled water (SDW). Seedlings were then grown at 22 ± 1°C and 120 µmol photons m^−2^. After 2 weeks, seedlings were flood inoculated as described previously (32). Plates were flooded with 40 ml of 10 mM MgCl_2_ containing 0.025% Silwett L-77 and ~5 × 10^5^ CFU *P. syringae* pv. tomato DC3000 and/or DC3000Δ*chp8* singly or together in a 1:1 ratio for 3 min and then drained. Seedlings were then grown for a further 4 days postinfection (d.p.i.). Seedlings were harvested at 1 and 2 d.p.i. in order to determine *in planta* bacterial cell counts and seedling chlorosis and quantify JA, SA, and ABA phytohormones. For *in planta* bacterial cell counts, seedlings were harvested from plates postinfection and surface sterilized in 70% ethanol for 1 min. Leaves were blotted dry and rinsed in SDW for 1 min before being homogenized in 500 µl of PBS. Serial dilutions were plated, and CFU per gram of plant determined. All *in planta* bacterial cell counts were performed in triplicate. To determine chlorosis levels, multiple seedlings were imaged postinfection. Representative images for those taken from each experimental group are shown. ABA, SA, and JA were extracted and quantified as described in reference 37 using seedling material as described in reference 80. Briefly, tissue was harvested and immediately frozen in N_2_. Samples were freeze-dried in a Heto Drywiner DW1.0-60e for 24 h. Samples were extracted in 394 µl of extraction solution composed of 25% methanol, 1% acetic acid in water. Internal standards were then added as follows: 2 µl jasmonic acid ([^13^C_2_]JA, 5 µg ml^−1^), 2 µl salicylic acid ([^2^H_4_]SA, 100 µM), and 2 µl abscisic acid ([^2^H_6_]ABA, 0.5 µg ml^−1^). A 3 mM tungsten bead was also added. Samples were placed in a Qiagen TissueLyser at 25.5 Hz for 1 min 50 s and incubated on ice for 30 min. Samples were centrifuged at maximum speed, and the supernatant removed. Samples were re-extracted using 400 µl of extraction buffer, and both extractions were pooled and transferred to vials for LC-MS/MS analysis. An injection volume of 50 µl was used. Analysis was performed on an Agilent 1100LC coupled to an Applied biosystems Q-TRAP LC-MS/MS system. Separation of molecules based on hydrophobicity was achieved using a Phenomenex Luna C_18_(2) column (100 mm by 2.0 mm by 3 µm) kept at 35°C. JA/SA/ABA ion pairs were monitored based on the following mass transitions: JA 209.2→59, [^13^C_2_]JA 211.2→61, SA 137.1→93, [^2^H_4_]SA 141.1→97, ABA 263.2→153, and [^2^H_6_]ABA 269.2→159. Data analysis was performed using Analyst, and the means determined based on 4 technical repeats are shown. Error bars denote standard errors of the means.

### Statistical analysis.

Statistical analysis was performed using GraphPad Prism software, version 6.

## SUPPLEMENTAL MATERIAL

Figure S1 Effect of HrpL on Chp8 promoter activity. Shown are the *chp8* promoter activities in response to plant cells in *P*. *syringae* pv. tomato DC3000, DC3000Δ*hrpL*, and DC3000Δ*hrpS*. Statistical analysis of P_*chp8*_ activity using unpaired *t* test gave results as follows (significant if *P* value is <0.05): DC3000 (plant cells) versus DC3000Δ*hrpL* (plant cells) was not significant, *P* = 0.2089 Download Figure S1, JPG file, 0.1 MB

Figure S2 In silico analyses of Chp8. Domain predictions using the Conserved Domain Database (CDD; NCBI) and multiple sequence alignments using MultAlin software (F. Corpet, Nucleic Acids Res. **16**:10881-10890, 1988, doi:10.1093/nar/16.22.10881) suggest that Chp8 contains a GGDEF and an EAL domain that are characteristic of diguanylate cyclases (DGC) and phosphodiesterases (PDE), respectively. The primary sequence of Chp8’s GGDEF domain was aligned with the known DGCs YcdT (*Escherichia coli*), YedQ (*E. coli*), PleD (*Caulobacter crescentus*), and YegE (*E. coli*). The primary sequence of Chp8’s EAL domain was aligned with the known PDEs YciR (*E. coli*), HmsP (*Yersinia pestis*), and BphG1 (*Rhodobacter sphaeroides*). Red letters, highly conserved amino acids; blue letters, less conserved amino acids; I site and A site, inhibitory and active sites of the GGDEF domain; green box, RXXD motif of the I site within the GGDEF domain (note that the RXXD motif of Chp8 is replaced by SXXV); blue boxes, signature GGDEF and EAL motifs (alanine substitution inactivates the respective domains) (S. L. Kuchma, M. Kimberly, K. M. Brothers, J. H. Merritt, N. T. Liberati, F. M. Ausubel, and G. A. O’Toole, J. Bacteriol. **189**:8165-8178, 2007, doi:10.1128/JB.00586-07) (note that both motifs are conserved in Chp8); yellow box, motif is highly conserved in active but degenerate in inactive PDEs (A. J. Schmidt, D. A. Ryjenkov, and M. Gomelsky, J. Bacteriol. **187**:4774-4781, 2005, doi:10.1128/JB.187.14.4774-4781.2005, and F. Rao, Y. Yang, Y. Qi, and Z.-X. Liang, J. Bacteriol. **190**:3622-3631, 2008, doi:10.1128/JB.00165-08) (note that this motif is also conserved in Chp8, indicating that the PDE domain of Chp8 is functional, in line with our phenotypic observations). Download Figure S2, JPG file, 0.6 MB

Figure S3 *P. syringae* pv. tomato DC3000 versus DC3000Δ*chp8 ex planta*. Shown are the outcomes of c-di-GMP, biofilm, and motility measurements of DC3000 versus DC3000Δ*chp8 ex planta*. Statistical analysis using unpaired *t* test gave results as follows (significant if *P* value is <0.05): c-di-GMP in DC3000 versus DC3000Δ*chp8* was not significant, *P* = 0.2693; biofilm of DC3000 versus DC3000Δ*chp8* was not significant, *P* = 0.0503. Download Figure S3, JPG file, 0.1 MB

Figure S4 Loading control for flagellin quantification. Shown is the loading control used for the quantification of flagellin levels in *P. syringae* pv. tomato DC3000Δ*chp8*/pSEVA and DC3000Δ*chp8*/pSEVA*chp8*_DGC_^+^_PDE_^−^ cells. The proteins were stained using the SYPRO Ruby protein stain (Molecular Probes). Fluorescence intensity of total protein loaded per lane was measured using the FLA-5000 imaging system (FujiFilm) in combination with the AIDA Image Analyzer software. Download Figure S4, JPG file, 0.1 MB

Figure S5 Phytohormones normalized to CFU/g plant. The phytohormone data were also expressed per CFU/g plant to obtain additional specific activity assessments, and these data again show differences in plant responses attributable to Chp8 function that are most evident at early time points and further emphasize that Chp8 has the strongest effect on SA levels. Statistical analysis using unpaired *t* test gave results as follows (significant if *P* value is <0.05): ABA, *P. syringae* pv. tomato DC3000 versus DC3000Δ*chp8* at 1 d.p.i. was not significant, *P* = 0.0502, and at 2 d.p.i. was not significant, *P* = 0.1324; SA, DC3000 versus DC3000Δ*chp8* at 1 d.p.i. was significant, *P* = 0.0015, and at 2 d.p.i. was significant, *P* = 0.0122; JA, DC3000 versus DC3000Δ*chp8* at 1 d.p.i. was not significant, *P* = 0.1591, and at 2 d.p.i. was not significant, *P* = 0.3862. Download Figure S5, JPG file, 0.1 MB

Table S1 Primers and plasmids used. Table S1, DOCX file, 0.1 MB.
