# Supplementary figures and images for: Chp8, a Diguanylate Cyclase from Pseudomonas syringae pv. Tomato DC3000, Suppresses the Pathogen-Associated Molecular Pattern Flagellin, Increases Extracellular Polysaccharides, and Promotes Plant Immune Evasion
Source: mBio. 2014 May 20;5(3):e01168-14. doi: 10.1128/mBio.01168-14 (PMC4030453; doi:10.1128/mBio.01168-14)

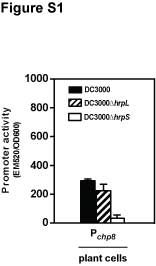

Supplement: Figure S1 — Effect of HrpL on Chp8 promoter activity. Shown are the chp8 promoter activities in response to plant cells in P. syringae pv. tomato DC3000, DC3000ΔhrpL, and DC3000ΔhrpS. Statistical analysis of Pchp8 activity using unpaired t test gave results as follows (significant if P value is <0.05): DC3000 (plant cells) versus DC3000ΔhrpL (plant cells) was not significant, P = 0.2089 Download [file mbo003141839sf01.jpg]

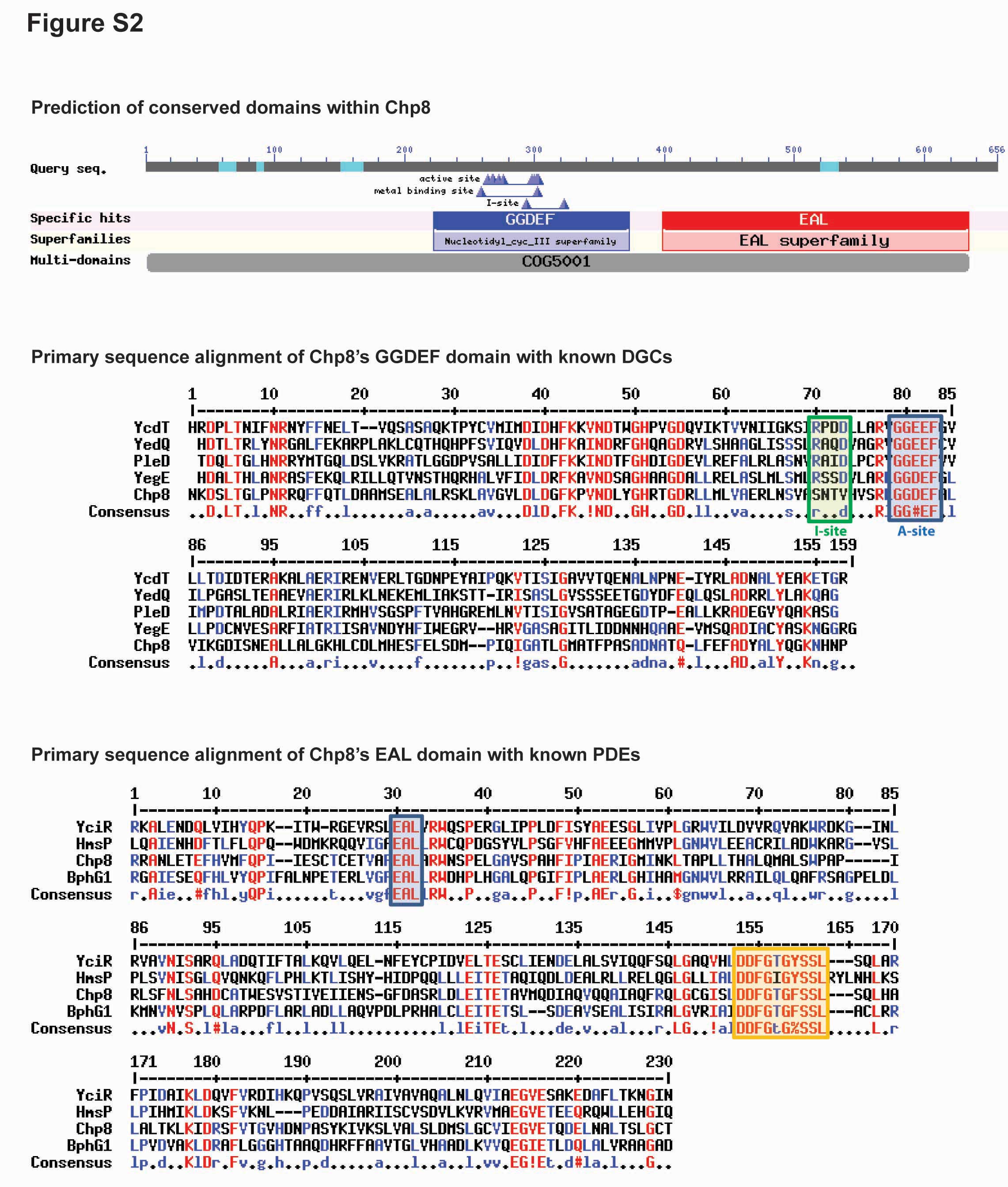

Supplement: Figure S2 — In silico analyses of Chp8. Domain predictions using the Conserved Domain Database (CDD; NCBI) and multiple sequence alignments using MultAlin software (F. Corpet, Nucleic Acids Res. 16:10881-10890, 1988, doi:10.1093/nar/16.22.10881) suggest that Chp8 contains a GGDEF and an EAL domain that are characteristic of diguanylate cyclases (DGC) and phosphodiesterases (PDE), respectively. The primary sequence of Chp8’s GGDEF domain was aligned with the known DGCs YcdT (Escherichia coli), YedQ (E. coli), PleD (Caulobacter crescentus), and YegE (E. coli). The primary sequence of Chp8’s EAL domain was aligned with the known PDEs YciR (E. coli), HmsP (Yersinia pestis), and BphG1 (Rhodobacter sphaeroides). Red letters, highly conserved amino acids; blue letters, less conserved amino acids; I site and A site, inhibitory and active sites of the GGDEF domain; green box, RXXD motif of the I site within the GGDEF domain (note that the RXXD motif of Chp8 is replaced by SXXV); blue boxes, signature GGDEF and EAL motifs (alanine substitution inactivates the respective domains) (S. L. Kuchma, M. Kimberly, K. M. Brothers, J. H. Merritt, N. T. Liberati, F. M. Ausubel, and G. A. O’Toole, J. Bacteriol. 189:8165-8178, 2007, doi:10.1128/JB.00586-07) (note that both motifs are conserved in Chp8); yellow box, motif is highly conserved in active but degenerate in inactive PDEs (A. J. Schmidt, D. A. Ryjenkov, and M. Gomelsky, J. Bacteriol. 187:4774-4781, 2005, doi:10.1128/JB.187.14.4774-4781.2005, and F. Rao, Y. Yang, Y. Qi, and Z.-X. Liang, J. Bacteriol. 190:3622-3631, 2008, doi:10.1128/JB.00165-08) (note that this motif is also conserved in Chp8, indicating that the PDE domain of Chp8 is functional, in line with our phenotypic observations). Download [file mbo003141839sf02.jpg]

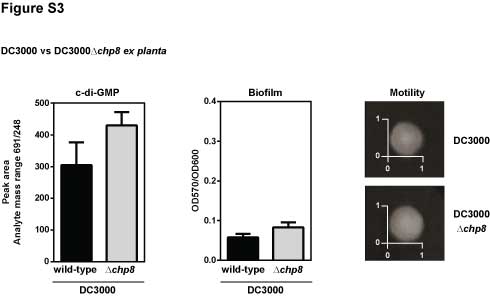

Supplement: Figure S3 — P. syringae pv. tomato DC3000 versus DC3000Δchp8 ex planta. Shown are the outcomes of c-di-GMP, biofilm, and motility measurements of DC3000 versus DC3000Δchp8 ex planta. Statistical analysis using unpaired t test gave results as follows (significant if P value is <0.05): c-di-GMP in DC3000 versus DC3000Δchp8 was not significant, P = 0.2693; biofilm of DC3000 versus DC3000Δchp8 was not significant, P = 0.0503. Download [file mbo003141839sf03.jpg]

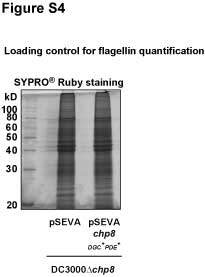

Supplement: Figure S4 — Loading control for flagellin quantification. Shown is the loading control used for the quantification of flagellin levels in P. syringae pv. tomato DC3000Δchp8/pSEVA and DC3000Δchp8/pSEVAchp8DGC+PDE− cells. The proteins were stained using the SYPRO Ruby protein stain (Molecular Probes). Fluorescence intensity of total protein loaded per lane was measured using the FLA-5000 imaging system (FujiFilm) in combination with the AIDA Image Analyzer software. Download [file mbo003141839sf04.jpg]

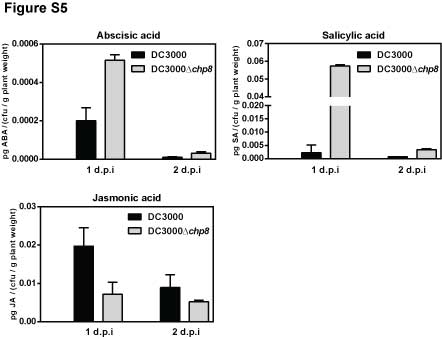

Supplement: Figure S5 — Phytohormones normalized to CFU/g plant. The phytohormone data were also expressed per CFU/g plant to obtain additional specific activity assessments, and these data again show differences in plant responses attributable to Chp8 function that are most evident at early time points and further emphasize that Chp8 has the strongest effect on SA levels. Statistical analysis using unpaired t test gave results as follows (significant if P value is <0.05): ABA, P. syringae pv. tomato DC3000 versus DC3000Δchp8 at 1 d.p.i. was not significant, P = 0.0502, and at 2 d.p.i. was not significant, P = 0.1324; SA, DC3000 versus DC3000Δchp8 at 1 d.p.i. was significant, P = 0.0015, and at 2 d.p.i. was significant, P = 0.0122; JA, DC3000 versus DC3000Δchp8 at 1 d.p.i. was not significant, P = 0.1591, and at 2 d.p.i. was not significant, P = 0.3862. Download [file mbo003141839sf05.jpg]
