# Supplementary material for: Chp8, a Diguanylate Cyclase from Pseudomonas syringae pv. Tomato DC3000, Suppresses the Pathogen-Associated Molecular Pattern Flagellin, Increases Extracellular Polysaccharides, and Promotes Plant Immune Evasion
Source: mBio. 2014 May 20;5(3):e01168-14. doi: 10.1128/mBio.01168-14 (PMC4030453; doi:10.1128/mBio.01168-14)
Supplement: Table S1 — Primers and plasmids used. [file mbo003141839st1.docx]

**Table S1: Primers and plasmids.**

| **Primer** | **Sequence (5’-3’)** | **Target** |
| --- | --- | --- |
| HrpSa | CTC CAG TGG TGG CAC CTT GTA CCT | flanking region upstream of *hrpS* |
| HrpSb | CCT ATA GTG AGT CGG ATC CCC TCC CAT GAC CCC CAG GAC ATC AAC | flanking region upstream of *hrpS*; with BamHI overlap |
| HrpSc | GGG GAT CCG ACT CAC TAT AGG TGC AAA GAC GCT GGA ACC GAT TCG | flanking region downstream of *hrpS*; with BamHI overlap |
| HrpSd | CGA TGC TGT TAG CCG ACT TGC CAT | flanking region downstream of *hrpS* |
| HrpLa | ACC GTC GGT GTA ATC CAG AAC CTG T | flanking region upstream of *hrpL* |
| HrpLb | CCT ATA GTG AGT CGG ATC CCC GCT GGG TTG AGT CGA GGA TCA CAA T | flanking region upstream of *hrpL*; with BamHI overlap |
| HrpLc | GGG GAT CCG ACT CAC TAT AGG CAA CCA CAC AGT TGC CAT CGC TCA | flanking region downstream of *hrpL*; with BamHI overlap |
| HrpLd | GCC GGG TTG GCG TTG ATG AAA TCC | flanking region downstream of *hrpL* |
| Chp8a | CAG TTC AGA CCC GAA ACT GGC GAC | flanking region upstream of *chp8* |
| Chp8b | CC TAT AGT GAG TCG GAT CCC CCG CAA GGC TTC TCT GTT ACC ACA GTC | flanking region upstream of *chp8*; with BamHI overlap |
| Chp8c | GGG GAT CCG ACT CAC TAT AGG CAG CGT GTA CGT GGG GTA AGA TGC | flanking region downstream of *chp8*; with BamHI overlap |
| Chp8d | AAA ATG TCG GCG TAA CGC AGG GTC | flanking region downstream of *chp8* |
| pChp8SphIF | CGG CGC ATG CGC GTC GGT AAC GGT TTA CGA CAA T | *chp8* promoter |
| pChp8BamHIR | CGG CGG ATC CCG CAA GGC TTC TCT GTT ACC ACA GT | *chp8* promoter |
| SphIphrpLGFPF | CGC GCA TGC GCC GGA TTA TGT CCG CTG AG | *hrpL* promoter |
| XbaIphrpLGFPR | CGC TCT AGA TAT AAA CGC AGA AAG GCC CAC C | *hrpL* promoter |
| EcoRIChp8sevaF | CGG CGA ATT CGC GTC GGT AAC GGT TTA CGA CA | *chp8* coding sequence |
| XbaIChp8sevaR | CGG CTC TAG ATC ATC AGA TAC GGC ACG CGG CTG CAT AAC CG | *chp8* coding sequence |
| Chp8_GGDEF::AAAAA_F | CAT GTG TCC CGA CTG GCC GCG GCC GCG GCC GCG CTG GTC ATC AAG | GGDEF motif coding sequence in *chp8* |
| Chp8_GGDEF::AAAAA_R | CTT GAT GAC CAG CGC GGC CGC GGC CGC GGC CAG TCG GGA CAC ATG | GGDEF motif coding sequence in *chp8* |
| Chp8_EAL::AAA_F | GAA ACG GTG GCT TTC GCG GCC GCG GCG CGT TGG AAC AGC | EAL motif coding sequence in *chp8* |
| Chp8_EAL::AAA_R | GCT GTT CCA ACG CGC CGC GGC CGC GAA AGC CAC CGT TTC | EAL motif coding sequence in *chp8* |
| **Plasmids** | **Features** | **Reference** |
| pFUSEhrpS | pGEM-T carrying the ≈600bp sequences that flank *hrpS* fused by the BamHI-containing overlap; Amp^R^ | This study |
| pFUSEhrpL | pGEM-T carrying the ≈600bp sequences that flank *hrpL* fused by the BamHI-containing overlap; Amp^R^ | This study |
| pFUSEchp8 | pGEM-T carrying the ≈600bp sequences that flank *chp8* fused by the BamHI-containing overlap; Amp^R^ | This study |
| pKOhrpS | pGEM-T derived allele exchange vector carrying the ∆*hrpS*::*nptII*FRT knockout allele; Amp^R^; Km^R^ | This study |
| pKOhrpL | pGEM-T derived allele exchange vector carrying the ∆*hrpL*::*nptII*FRT knockout allele; Amp^R^; Km^R^ | This study |
| pKOchp8 | pGEM-T derived allele exchange vector carrying the ∆*chp8*::*nptII*FRT knockout allele; Amp^R^; Km^R^ | This study |
| pBBR1-P*_chp8_*-*gfp* | pBBR1MCS-4 (70) carrying the *chp8* promoter fused to *gfpmut3* including the rbs30 ribosome binding site and a transcriptional terminator (71) | This study |
| pBBR1-P*_hrpL_*-*gfp* | pBBR1MCS-4 (70) carrying the *hrpL* promoter fused to *gfpmut3* including the rbs30 ribosome binding site and a transcriptional terminator (71) | This study |
| pSEVAchp8_DGC_^+^_PDE_^+^ | pSEVA224 (73) carrying the coding sequence for wild-type Chp8 | This study |
| pSEVAchp8_DGC_^+^_PDE_^-^ | pSEVA224 (73) carrying the coding sequence for Chp8 (EAL::AAA) | This study |
| pSEVAchp8_DGC_^-^_PDE_^+^ | pSEVA224 (73) carrying the coding sequence for Chp8 (GGDEF::AAAAA) | This study |
